# Effects of chitosan on hematological parameters and stress resistance in rainbow trout (*Oncorhynchus mykiss*)

**Published:** 2012

**Authors:** Saeed Meshkini, Ali-Akbar Tafy, Amir Tukmechi, Farhad Farhang-Pajuh

**Affiliations:** 1 *Department of food hygiene and Quality Control, Faculty of Veterinary Medicine and Artemia & Aquatic Animals Research Institute, Urmia University, Urmia, Iran; *; 2 *Department of Fisheries, Faculty of Natural Resources, Urmia University, Urmia, Iran; *; 3 *Department of Biotechnology and Pathobiology, Artemia and Aquatic Animals Research Institute, Urmia University, Urmia, Iran; *; 4 *Department of Parasitology, Faculty of Veterinary Medicine, Urmia University, Urmia, Iran.*

**Keywords:** Rainbow trout, Chitosan, Hematological parameters, Stress resistance

## Abstract

The aim of our study was to evaluate the effects of chitosan as immune stimulator on some hematological parameters and stress resistance in rainbow trout. Nine hundred rainbow trout (with initial body weight of 25 ± 0.1 g) were obtained from a local farm and acclimated to the laboratory conditions for one week. After that fish were randomly divided into four groups in three replicates. Each group received chitosan in diet at four concentrations as 0 (Control), 0.25, 0.5 and 1 percent chitosan, respectively. The trial was conducted for 8 weeks then feeding with chitosan stopped for 3 weeks later and during this time all fish were feed by control diet. The sampling was conducted to assay the hematological parameters of all groups every two weeks. In this study we assayed the resistance of fish against some environmental stresses immediately after changing the diet to the control. The results showed that using 0.25 percent chitosan in trout diets had a significant effect (*P *< 0.05) on hematological indices and stress resistance of rainbow trout in comparison the control group. Serum glucose level was higher in all treatment than control without any significance difference (*P *< 0.05). Based on the obtained results it concluded that the adding chitosan at 0.25 percent into the diet could enhance the hematological parameters and resistance against some environmental stresses in rainbow trout.

## Introduction

Fish in production facilities are exposed to stress conditions, diseases and deterioration of the environmental conditions that could cause serious economic losses.^1^ Currently, fish are protected from infectious diseases by vaccination or chemotherapeutic treatment. However, due to extensive use of the chemotherapeutic agents, the emergence of antimicrobial resistance among pathogens and the associated environmental hazards have been well documented. Therefore, several alternative strategies such as the use of different immune stimulators have been proposed.^2^ The use of immune stimulants in fish could improve fish resistance against unfavorable environmental conditions and pathogenic agents compared with other treatment methods.^2^ Chitosan is produced commercially by deacetylation of chitin, which is the structural element in the exoskeleton of crustaceans (such as crabs and shrimps) and cell walls of fungi. On average, the molecular weight of commercially produced chitosan is between 3800 to 20,000 Daltons. The amino group in chitosan has a pKa value of ~ 6.5, which leads to acidic to neutral solution with a charge density dependent on pH. This makes chitosan water soluble and a bio-adhesive which readily binds to negatively charged surfaces such as mucosal membranes. Chitosan enhances the transport of polar drugs across epithelial surfaces, and is biocompatible and biodegradable. Purified qualities of chitosan are available for biomedical applications.^3^

Chitosan and its derivatives such as trimethylchitosan (where the amino group has been trimethylated) have been used in non-viral gene delivery. Trimethylchitosan, or quaternised chitosan, has been shown to transfect breast cancer cells; with increased degree of trimethylation increasing the cytotoxicity and at approximately 50% trimethylation the derivative is the most efficient at gene delivery.^4^ It is also a natural polymer with different physical and chemical properties of chitin.^5^ chitosan have growth and immune stimulating properties in aquatic animals with solubility than chitin in water and other polar solvents.^6^^,^^7^ Previous studies showed that chitosan has immune stimulating properties in different species of fish.^8^^-^^12^

Based on our knowledge; the effects of chitosan on hematological indices and stress resistance have not been studied in rainbow trout. Thus, the aim of this study was to evaluate the effects of dietary chitosan on some blood parameters and stress resistance in rainbow trout. 

## Materials and Methods

**1. Fish and husbandry conditions. **In this study, 900 fish with a mean weight of 25 ± 0.1 g were obtained from a local farm in Urmia, Iran and transferred to “Artemia and Aquatic Animals Research Institute”. The fish were immediately disinfected with 3% sodium chloride for 5 min and acclimatized to the laboratory conditions for one week. Fish were randomly distributed into each of 12 PVC tanks (300 liter capacity) filled with 150 liters of water. Each tank was continuously supplied with aerated free-flowing dechlorinated fresh water with the flow rate set at 3.5 liters per second, water temperature 14 ± 1 °C and dissolved oxygen 8.5 ppm.

**2. Diet preparation and feeding trial. **The chemical composition of chitosan (Aminolabs^®^; Award, USA) in this study has been characterized as white yellowish powder, 34.90% humidity, 0.75 ash, 91.09 degree of deacetylation, and 0.61 g mL^-1^ of density. Commercial fish feed (Faradaneh Co., Iran) was used during the study with 90% dry Mater, 38% crude protein, 16% crude lipid, 10% ash, 3% fiber and 1.20% phosphorous in its composition. 

Three experimental diets were formulated to be supplemented at 0.25, 0.50, and 1.00 g kg^-1^ of diets. One percent acetic acid was used for the chitosan to the fish feed. In control group, acetic acid was also added to fish feed for making the situation the same as treatment groups. After spraying the chitosan on commercial feed, pellets were dried at room temperature for 2 hours (h) and then they were stored at 4 °C for further use. Each diet was fed to triplicate tanks three times daily for a period 8 weeks.^13^ After feeding trial, diets were replaced by normal pelleted diet without any chitosan.

**3. Blood sampling, preparation and measurement of serum blood parameters. **On weeks 0, 2, 4, 6 and 8 during the feeding trial and 3 weeks after finishing the feeding trial, 5 fish from each tank were sampled to determine the hematological parameters. Blood was sampled from the caudal vein after euthanasia by immersion in solution containing clove powder^14^; 200 mg L^-1^. The blood samples were divided into two aliquots; one aliquot was transferred into an Eppendrof tube and allowed to clot at room temperature for 1 h. Then, samples were kept at 4 °C for 5 h. The sera were separated by centrifugation (1500 *g* for 5 min at 4 °C). The sera samples were used for assaying the glucose level and stored at -80 °C until required. The other aliquot was mixed with heparin and used for counting red blood cells (RBCs) and white blood cells (WBCs). All blood cells were counted under a light microscope using Neubauer hemocytometer after dilution with phosphate-saline. Differential leukocyte counts (neutrophil, lymphocyte, and monocyte) were determined using Giemsa staining method of blood smears under a light microscope. Cells were identified on the basis of morphology and cell ultra-structure as documented in previous study.^15^ Also, serum glucose level was measured using a commercial kit based on the hexo-kinase-glucose-6-phosphate dehydrogenase method (Man Co., Iran).

**4. Stresses. **At week 8 and 11, 30 fish from each dietary treatment (10 fish per each tank) were subjected to some environmental stresses as below: 

**A) Hypoxic stress. **Hypoxic stress was induced according to a previous study.^16^ Briefly, 30 sampled fish were divided into two subgroups (15 fish) and exposed to hypoxia for 6 and 9 min, respectively. Then fish of each subgroup were placed in two separate tanks containing 90 liters of aerated flow-through well water. All fishes were monitored for 1 h and dead fish were removed. Then, mortality rate was recorded for each subgroup.

**B) Thermal stress. **Thermal stress was done in two 90 liter tanks with water temperature of each tank maintained at 8 and 11 °C, respectively. Fifteen fish were placed in each tank for 48 h and mortality was recorded every 6 h.

**C) Salinity stress. **15 fish were placed in two 90 liter tanks each with 25 and 35 ppt salinity equipped with continuous aeration. The stress lasted for 48 hours and mortality was recorded every 6h.

**5. Statistical analysis. **All the measurements were made in triplicates. The results were subjected to analysis of variance (ANOVA) followed by least significant differences Tukey test. Correlation coefficients were considered significant when *P* < 0.05.

## Results

**Fig. 1:**
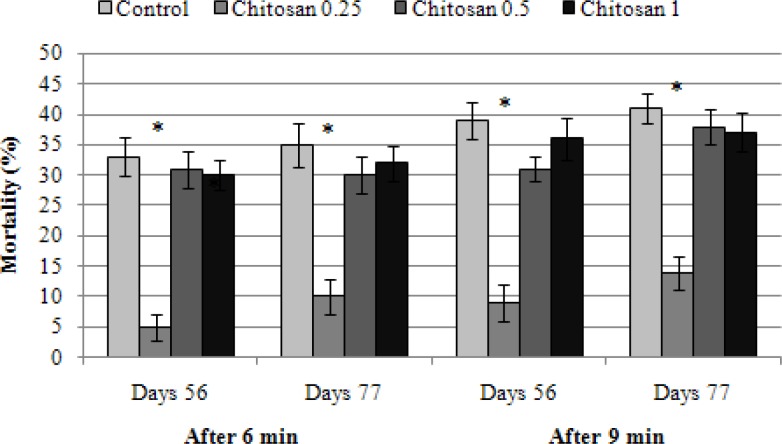
The mortality rate of rainbow trout fed with different concentrations of chitosan in low dissolved O_2_ after 6 and 9 min

**Fig. 2:**
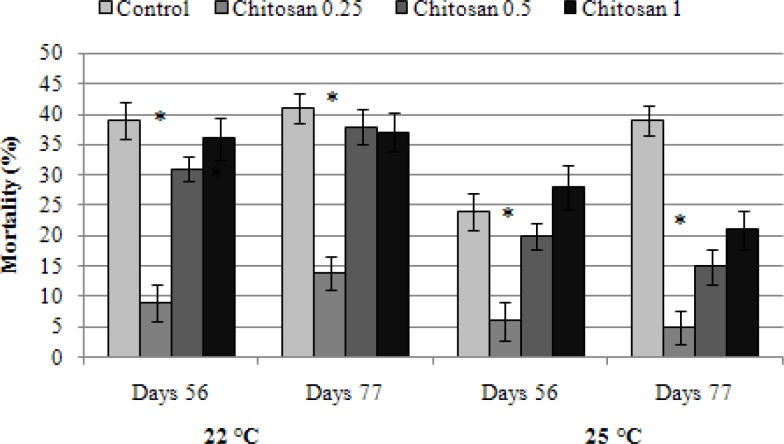
The mortality rate of rainbow trout fed with different concentrations of chitosan in thermal stress.

**Fig. 3:**
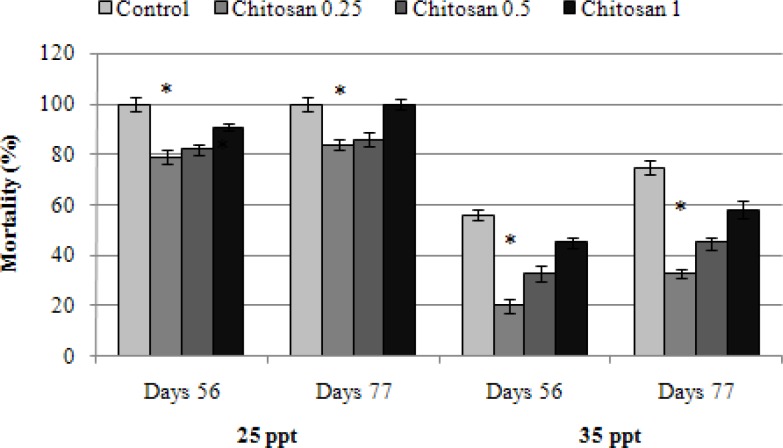
The mortality rate of rainbow trout fed with different concentrations of chitosan in salinity stress.

In Table 1 hematological parameters of fish fed with different concentrations of chitosan are shown. During the trial serum glucose level was higher in all treatment groups that received chitosan than the control group. The second treatment (T2) significantly (*P *< 0.05) had increased total white blood cells count (16838.00 ± 1608.61 cells per μL), lymphocyte (95.33 ± 1.15) and neutrophil (1.33 ± 1.15) percentage after week 8 compared to other group. The dietary supplementation with 0.25% chitosan led to a significant reduction in mortalities after environmental stresses including changes in water temperature, salinity and oxygenation (Figs. 1, 2, and 3).

**Table 1:** Hematological parameters in rainbow trout fed with different chitosan concentrations

**Blood Factors**	**Time** **(Week)**	**T1** **(Control)**	**T2** **(0.25% chitosan)**	**T3** **(0.50% chitosan)**	**T4** **(1.00% chitosan)**
**Total WBC** **(cells per μL Blood)**	Initial day	10253.33±1003.82^a^	11097.67±766.73^a^	10872.67±992.72^a^	10477.00±327.88^a^
	Week 2	10215.67±869.81^b^	13902.33±1543.44^a^	12782.67±253.72^a^	10669.00±741.46^b^
	Week 4	11416.33±518.24^b^	14616.00±1479.43^a^	13576.33±583.83^a^	11032.33±767.61^b^
	Week 6	12199.00±417.60^b^	15357.67±1433.09^a^	14068.33±533.01^a^	11987.00±578.04^b^
	Week 8	13452.33±840.10^b^	16838.00±1608.61^a^	14822.33±916.88^ab^	12915.67±855.27^b^
	Week 11	14241.67±1120.08^b^	18263.33±1301.40^a^	15938.00±1130.71^b^	14189.00±722.54^b^
**Lymphocyte (%)**	Initial day	84.67±1.52^a^	86.00±1.00^a^	84.66±1.53^a^	84.00±3.00^a^
	Week 2	85.67±0.58^ab^	88.33±1.53^a^	86.00±2.00^ab^	85.00±2.00^b^
	Week 4	86.67±1.53^b^	92.00±1.00^a^	88.67±2.52^ab^	86.67±2.08^b^
	Week 6	86.33±0.57^c^	94.00±1.00^a^	90.33±2.52^b^	88.33±1.53^bc^
	Week 8	86.33±1.15^c^	95.33±1.15^a^	91.00±2.64^b^	88.67±1.15^bc^
	Week 11	87.00±1.73^b^	94.00±2.00^a^	90.00±3.61^ab^	87.33±3.05^b^
**Monocyte (%)**	Initial day	2.67±0.58^a^	2.67±1.15^a^	2.67±1.15^a^	2.67±1.53^a^
	Week 2	2.67±1.53^a^	2.67±0.58^a^	2.67±1.53^a^	2.67±0.58^a^
	Week 4	3.00±0.00^a^	3.33±0.58^a^	2.67±1.53^a^	2.67±1.15^a^
	Week 6	2.67±0.58^a^	2.67±1.15^a^	3.33±0.58^a^	2.67±0.58^a^
	Week 8	2.67±1.53^a^	3.00±1.73^a^	2.67±1.15^a^	3.33±1.15^a^
	Week 11	2.67±1.15^a^	2.67±1.15^a^	3.33±1.15^a^	2.67±1.53^a^
**Neutrophil (%)**	Initial day	7.33±0.58^a^	6.67±1.15^a^	7.33±0.58^a^	7.33±2.52^a^
	Week 2	7.00±0.00^a^	7.33±1.15^a^	6.00±1.72^a^	7.33±1.53^a^
	Week 4	7.33±0.58^a^	4.67±1.15^b^	6.00±1.00^ab^	7.33±2.08^a^
	Week 6	7.67±1.15^a^	3.33±1.53^c^	4.67±1.53^bc^	6.00±0.00^ab^
	Week 8	7.67±0.58^a^	1.33±1.15^c^	4.33±2.08^b^	6.33±1.15^ab^
	Week 11	7.33±0.58^a^	2.00±2.00^b^	5.00±1.00^a^	6.67±0.58^a^
**Eosinophil (%)**	Initial day	5.33±1.15^a^	4.67±1.52^a^	5.67±0.58^a^	6.00±2.00^a^
	Week 2	4.67±1.15^a^	1.67±0.58^a^	5.33±1.52^a^	5.00±1.00^a^
	Week 4	3.00±2.00^a^	0.00±0.00^a^	2.67±0.58^a^	3.33±0.58^a^
	Week 6	3.33±1.53^a^	0.00±0.00^a^	1.67±1.15^a^	3.00±2.00^a^
	Week 8	3.33±1.15^a^	0.67±0.58^a^	2.00±2.00^a^	1.67±1.15^a^
	Week 11	1.67±1.15^a^	0.00±0.00^a^	0.67±0.58^a^	2.00±0.00^a^

abc The same superscript alphabets in the same raw are not significantly different at *P* < 0.05.

**Table 2:** Serum glucose level (mg dL^-1^) in rainbow trout fed with different chitosan concentrations

**Treatments**	**Weeks**					
	**Initial day**	**Week 2**	**Week 4**	**Week 6**	**Week 8**	**Week 11**
**T1 (Control)**	8.50±1.11^a^	9.69±0.59^b^	9.82±1.27^a^	9.33±3.97^a^	9.89±0.69^c^	9.99±2.01^c^
**T2 (0.25% chitosan)**	8.38±2.60^a^	12.50±1.38^a^	12.84±0.75^a^	14.00±2.02^a^	17.61±2.08^a^	16.91±1.65^a^
**T3 (0. 50% chitosan)**	8.28±3.09^a^	11.29±1.05^ab^	11.56±5.01^a^	12.62±1.18^a^	14.48±0.77^b^	13.61±2.04^b^
**T4 (1.00% chitosan)**	8.43±0.97^a^	9.96±0.51^b^	10.39±0.98^a^	10.65±1.54^a^	11.25±1.00^c^	10.29±0.94^c^

abc The same superscript alphabets in the same raw are not significantly different at *P* < 0.05.

## Discussion

To date, many studies have been done about the use of immune stimulants in aquaculture. For example, some substances such as glucan,^17^^,^^18^ lactoferin,^19^ levamisole,^20^ FK- 565,^21^ chitin and chitosan,^22^^,^^23^ and EF-203^21^ have success-fully enhanced the fish immune response. Immune stimulators could increase serum lysozyme activity,^18^^,^^24^ alternative complement activity^24^ and total antibody production^17^ in fish. Immune stimulation improves fish resistance against undesirable conditions such as environmental stresses and diseases. We study the effect of chitosan as a natural substance on hematological parameters and stress resistance in rainbow trout. For this purpose, the number, type and percentage of WBC, blood glucose level and fish resistance against salinity, temperature and hypoxia were assayed. 

Lymphocytes have a key role in specific defense mechanisms (acquired immunity) known as an important immune component cells. Lymphocytes are found in blood, lymphatic organs and other tissues and contribute in antibody production and phagocytic activity.^25^ On the other hand, monocyte and granulocytes (neutrophil, eosinophil and basophil) are involved in non-specific cellular immunity of fish. The major act of monocyte is phagocytosis but more functions are attributed to the neutrophil. For example, they kill pathogens with their enzymes and active oxygen radicals; they also participate in phagocytic activity.^26^ In the present study, chitosan (0.25% of diet) significantly increased the number of white blood cells in rainbow trout for a period of 8 weeks (Table 2). Also, lymphocytes percentage significantly increased in the fish group that received 0.25% chitosan with their diet compared with other treatments and control. The results from our study showed that the monocyte and eosinophil number did not increase in any groups of fish fed with chitosan. The percentage of neutrophil decreased in fish received 0.25% chitosan (Table 2). Other studies have showed lymphocytes percentage and their functions could be improved by immuno-stimulants.^27^ Levamisole, alginic acids, bovine lactoferin, vitamin C vitamin E and chitin significantly have positive effects on the white blood cell especially on the lymphocytes population of fish and shrimp.^2^^,^^28^^-^^30^ Our results are in agreement with above studies and it has demonstrated that chitosan could stimulate the white blood cells function and rainbow trout immune system. Also, Faghani *et al.,* reported that oral administration of alginic acid in rainbow trout increased the number of white blood cells and fish resistance against environmental stresses.^31^

The fish received 0.25% chitosan in their diet significantly had less mortality rate at hypoxic stress than other groups and the control (Fig. 1). The same results were also found with salinity and temperature stresses (Figs. 2 and 3). Our results indicated that chitosan could increase fish survival against stresses.

Booniaratpalin *et al.* reported that when *Peneaus monodon* (Black tiger shrimp) fed with dietary peptido-glycan showed high survival rate against low dissolved oxygen, salinity and other stresses than the control.^32^ Based on these studies and obtained results of our work, we conclude that improvement of hematological parameters and resistance quality of rainbow trout could be due to an elevated number of total white blood cells. 

In aquaculture, concentrations and duration of immune stimulators application is important to obtain the best results. Robertsen *et al*. reported that the macrophage activity of the fish which injected with 0.1-1 µg mL^-1^ of glucan was higher than the 10 and 50 µg mL^-1^.^33^ They showed 10 µg mL^-1^ of glucan did not have any effect on macrophage activity, whereas 50 µg mL^-1^ of glucan had reveres effects on macrophage and lymphocytes activity. Our results agree with mentioned findings because chitosan in 0.25% of rainbow trout diet had positive effects on immune system than 0.5% and 1%.

On the other hand, Matsuo and Miyazano showed a period of 56 days administration of peptidoglycan in rainbow trout diet had no any effects on hematological indices, immune system and protection against *Vibrio anguillarum*,^34^ whereas peptidoglycan could support that in a short time (28 days). We indicate that chitosan can increase rainbow trout resistance against environmental conditions and improve hematological parameters for 56 days. 

In conclusion, it seems that dietary administration of 0.25% chitosan for up 56 days could enhance rainbow trout hematological parameters and resistance against low oxygen, salinity and temperature stresses.
